# Symptomatic Cervical Tumoral Calcinosis due to Cosmetic Body Contouring Mineral Oil Injections

**DOI:** 10.7759/cureus.11743

**Published:** 2020-11-28

**Authors:** Julie L Chan, Robin Babadjouni, Wendy Sacks, Serguei I Bannykh, Alexander Tuchman

**Affiliations:** 1 Neurosurgery, Cedars-Sinai Medical Center, Los Angeles, USA; 2 Endocrinology, Cedars-Sinai Medical Center, Los Angeles, USA; 3 Pathology and Laboratory Medicine, Cedars-Sinai Medical Center, Los Angeles, USA

**Keywords:** tumoral calcinosis, foreign body injections, body contouring, cervical stenosis

## Abstract

Tumoral calcinosis (TC) is an uncommon disease that has been linked to familial genetic mutations but can often be due to secondary causes such as chronic renal failure and hyperparathyroidism. There are rare instances of tumoral calcinosis induced by foreign body injections, often for cosmetic purposes. Here we describe operative management of spinal cord compression due to mineral oil injection induced tumoral calcinosis. A 54-year-old transgender female presented with signs of myelopathy so severe that she had become wheelchair bound. Labs demonstrated hypercalcemia and imaging of the neuroaxis revealed significant calcification resulting in cervicothoracic and lumbar central canal stenosis. Given symptomatic cervical spinal cord compression, she was taken to the OR for urgent laminectomy and decompression. Postoperatively, she recovered well and was ambulating independently by postoperative day (POD) 9. This is the first reported case of localized mineral oil injections causing distant calcification with subsequent symptomatic cord compression requiring operative intervention.

## Introduction

Tumoral calcinosis (TC) was first described in 1948, and since then a number of underlying etiologies have been discovered [1]. The primary inherited forms related various gene mutations are the most common, however, there are also secondary forms associated with chronic renal failure leading to secondary or tertiary hyperparathyroidism. The cause of these calcific growths is variable, and recent literature has even described an association between cosmetic paraffin oil, mineral oil, or silicone injections and hypercalcemia, local lipogranulomas, or TC [2-4]. The typical gross description is a soft lesion with lobulated calcifications typically found at the joints. Although TC is most often found in the extremities, there are a growing number of cases of calcified tumors due to various etiologies in the spine and even the spinal canal [5-10]. Recent reports have noted that the clinical presentation may be subacute or even acute in nature as described by Al Sukaini et al. in a case report and review of five cases in the literature [11]. While TC of the spine has been reported, and there are reports of foreign body local inflammatory reactions and systemic hypercalcemia, none have linked these seemingly disparate pathologies. Here we present the first known report of mineral oil injections for body contouring causing hypercalcemia and distant calcification of the cervical spine with symptomatic stenosis.

## Case presentation

History

 A 54-year-old transgender female presented to the ED with five months of progressive gait instability resulting in debilitation so severe that she had become wheelchair dependent. She also reported lumbar back pain and right lower extremity radiculopathy. Her history was notable for HIV+ intermittently compliant on antiretroviral medications, hypercalcemia, and chronic kidney disease. She also had undergone two body contouring procedures with mineral oil injections of her buttocks and thighs 20 years prior in the process of transitioning to a woman. Physical exam revealed right lower extremity paresis. In her right lower extremity she had 3/5 strength in dorsiflexion and 4/5 throughout the rest of the leg. There was decreased sensation in bilateral lower extremities with 4+ patellar deep tendon reflexes and ankle clonus. Her buttocks and posterior thighs demonstrated diffusely hard darkened skin, and a 10 cm x 10 cm well-circumscribed midline nodule at the level of the sacrum. A separate but similar 5 cm x 5 cm nodule was also noted in the lumbar area. Laboratory results demonstrated hypercalcemia (13.2 mg/dL), depressed parathyroid hormone (PTH) (4.8 pg/mL), slightly elevated parathyroid hormone-related protein (PTHrp) (5.0 pmol), and elevated Vitamin D 1,25 OH (248 pg/mL).

**Figure 1:**
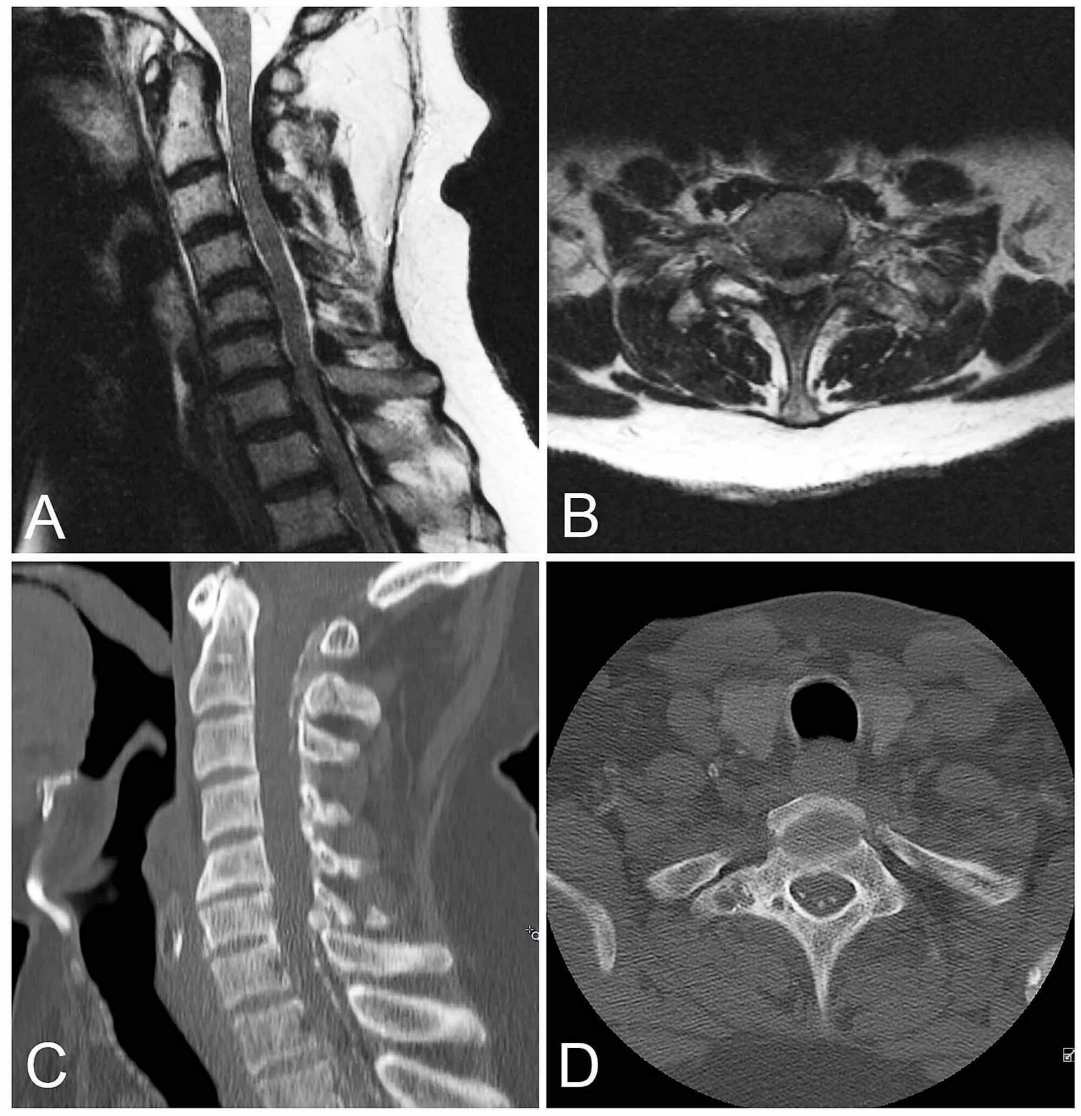
Cervical spine MRI and CT demonstrating spinal stenosis. Sagittal and axial T2 weighted MRI (A, B) of the cervical spine demonstrate severe central canal stenosis worse at C6-T1 causing mild T2 cord signal change. Sagittal and axial CT of the cervical spine (C, D) demonstrate extensive posterior calcified lesions throughout the cervical canal.

The MRI of the neuroaxis demonstrated a small amount of posterior epidural fluid from C7-T3, multilevel degenerative changes of the cervical spine, and severe spinal canal stenosis from C7-T1 resulting in cord compression and myelomalacia (Figure 1A,B). CT of the cervical spine re-demonstrated the cervical central canal stenosis, highlighting a circumferential calcifying process involving the ligaments and lamina (Figure 1C,D). Given her progressive weakness and clear cord compression due to these calcified lesions, she was taken to the OR in an urgent fashion.

Operative procedure

She was placed prone on a Jackson table in Mayfield pins. A midline cervicothoracic incision was made, and the posterior elements exposed. A standard midline laminectomy was performed, removing the entire lamina of C7 and T1, with extension to the inferior portion of C6, and superior portion of T2. The lamina was adherent to an epidural collection of calcified phlegmon which was adherent to the dura. This was carefully excised using a combination of curettes and Kerrison rongeurs until normal appearing dura was visualized at the margins of the laminectomy, and the spinal cord adequately decompressed. The phlegmon was sent to pathology and microbiology. The wound was irrigated, hemostasis achieved, and closed in layers with a drain left in place. 

Postoperative course

**Figure 2:**
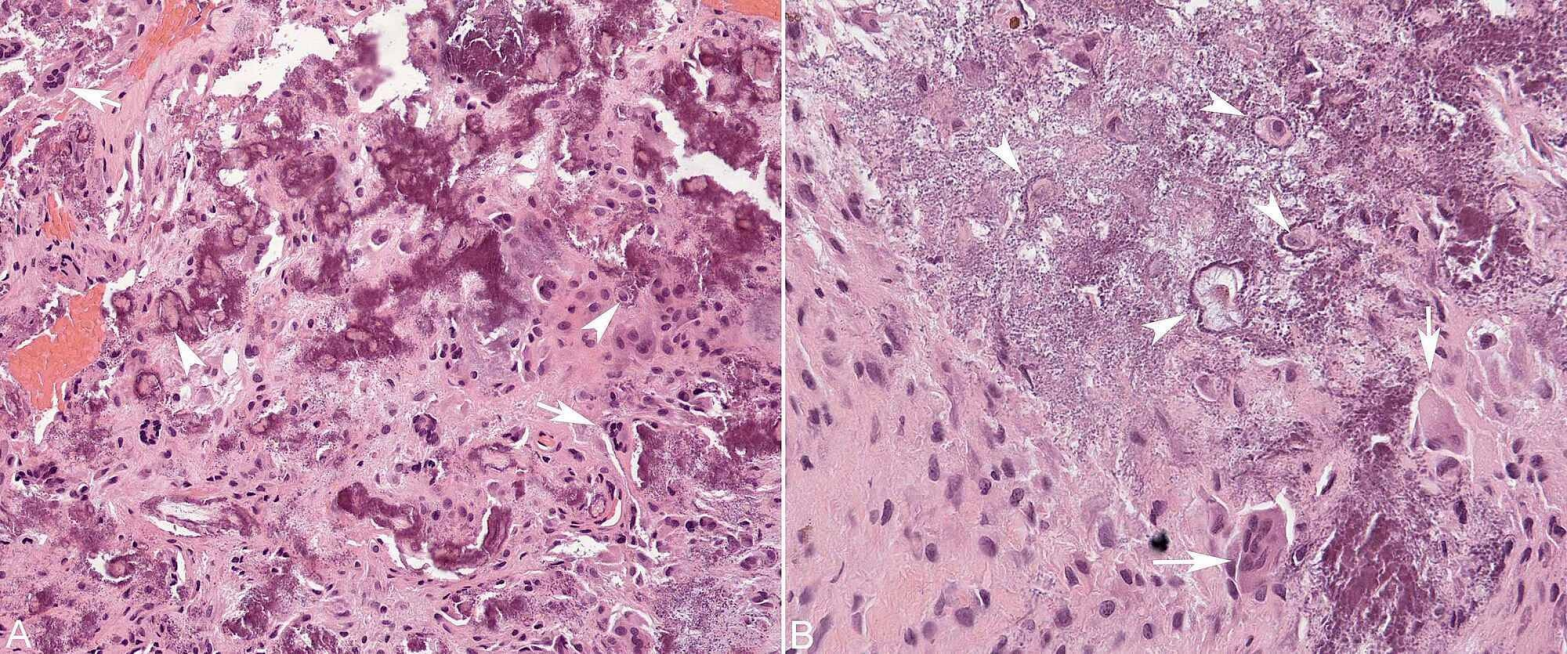
Histological examination of epidural mass. Hematoxylin and eosin stained section of the epidural mass discloses lacy "dystrophic" calcification of extracellular matrix (purple deposits) as well as lacunes of chondrocytes (white arrowheads). There is foreign body-type giant cell granulomatous reaction (white arrows) and exuberant proliferation of synoviocytes. Original magnifications: 200x (A), 400x (B).

Immediately after surgery, she demonstrated significant improvement in right lower extremity strength, numbness, and gait. By postoperative day (POD 9) she regained full strength with significant improvement in deep tendon reflexes and clonus. In addition to cervical decompression, she also underwent a lymph node biopsy. Final spinal pathology demonstrated fibrous connective tissue with nonpolarizable calcifying crystalline material associated with foreign body-type granulomatous reaction, and calcification consistent with TC (Figure 2). The lymph node biopsy demonstrated lipogranuloma. She was treated with zoledronate and hydroxychloroquine and on POD 25, she was discharged ambulating independently with strict instructions for outpatient calcium level follow up.

## Discussion

Tumoral calcinosis is an uncommon entity, and a rare finding in the spine. The largest series describes 21 cases of nearly missed diagnosis due to the variable presentation on MRI, CT, and XR [12]. Familiarity with the disease process is the key first step in diagnosis, and prior studies have noted the importance of multimodal imaging and detailed history. Al-Sukaini et al. reported the acute decompensation of a patient with cervical TC and discussed 10 symptomatic and nonsymptomatic reports of TC in the spine of adult patients [11]. The importance of a detailed history is supported by the literature which notes many patients with spinal TC have a history of rheumatologic disease such as Raynaud’s, scleroderma, and/or rheumatoid arthritis [13-15] while other cohorts demonstrated renal failure and/or hyperparathyroidism [7, 16-17].

 Nonparathyroid hypercalcemia has been recently linked to exogenous foreign body injections with silicone, paraffin oil, and mineral oil which may be used during cosmetic procedures as dermal fillers for body contouring [2, 4]. Local lipogranuloma reactions are described, with the most common locations being at the injection site such as the buttock, breast, lower extremities, or face [18-19]. Local calcifications are consistent with our patient who demonstrated hardened areas within her hips and buttocks near the initial mineral oil injection sites, as well as lipogranuloma of the groin lymph node. It is important to recognize that the downstream side effects of foreign body injections are not always immediate, but may occur in an indolent fashion as was the case with our patient [3, 20].

 While hypercalcemia and TC as a reaction to cosmetic injections has been linked to local inflammatory and calcific reactions, this is the first report of symptomatic TC of the spine due to physically distant cosmetic mineral oil injections. Other causes of this calcification process include infectious and malignant etiologies, neither of which were likely causes in our patient. Most likely, the body contouring mineral oil injections induced a granulomatous reaction causing systemic hypercalcemia and subsequent TC. This is supported by abnormally high levels of Vitamin D 1,25 OH, moderate hypercalcemia, and low PTH. The mechanisms that predispose the spinal epidural space to the development of TC in our patient are obscure. One possibility is retrograde lymphatic spread of the local mineral oil injections to the epidural space, altering the intracellular lipid content and subsequent abnormal response to hypercalcemia. It is also possible that lymphatic spread of the mineral oil directly induced kidney damage.

## Conclusions

We present the first reported case of compressive TC in the cervical spine due to cosmetic injections of mineral oil causing granulomatous reaction and hypercalcemia. A thorough history, extensive medical workup, and careful evaluation of imaging are key elements in obtaining an accurate diagnosis to optimize the outcome of these rare cases.
